# Supplementary material for: Synaptically silent sensory hair cells in zebrafish are recruited after damage
Source: Nat Commun. 2018 Apr 11;9:1388. doi: 10.1038/s41467-018-03806-8 (PMC5895622; doi:10.1038/s41467-018-03806-8)
Supplement: Supplementary file 1 — Supplementary Information [file 41467_2018_3806_MOESM1_ESM.pdf]

Supplementary Information for, "Synaptically silent sensory hair cells in zebrafish are recruited after damage"

Qiuxiang Zhang, Suna Li, Hiu-Tung C. Wong, Xinyi J. He, Alisha Beirl, Ronald S. Petralia, Ya-Xian Wang, and Katie S. Kindt

Supplementary figures and legends

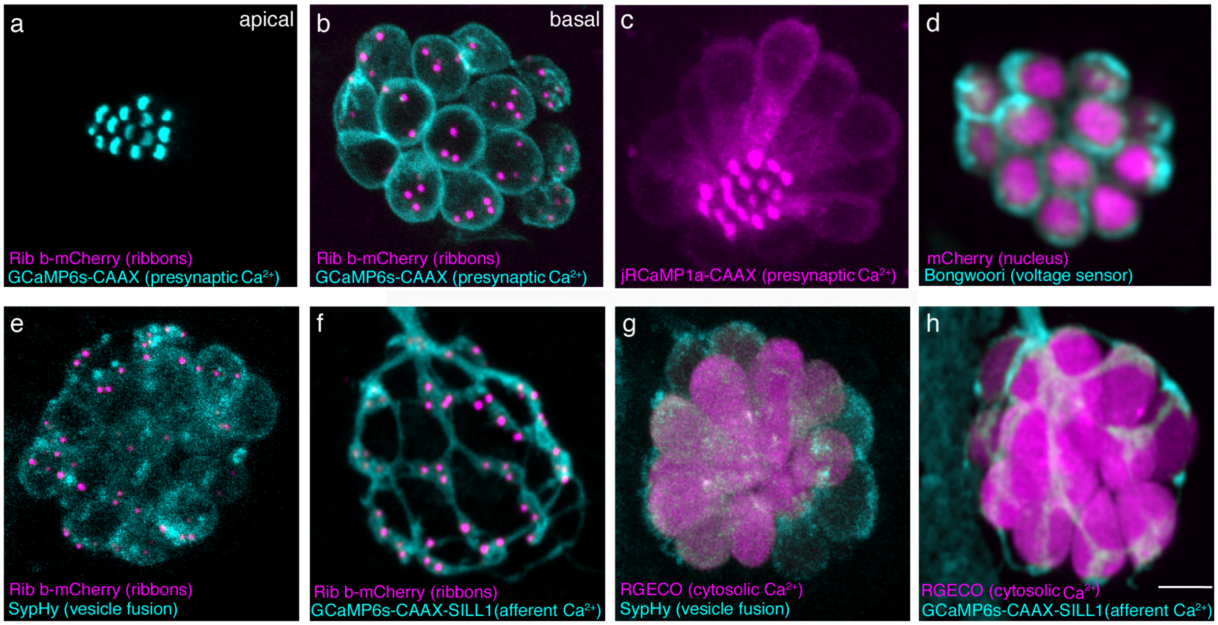

Supplementary Figure 1. **Zebrafish transgenic lines for functional imaging.** (a-b) Top-down view of a double transgenic neuromast expressing a membrane localized Ca^{2+} indicator, GCaMP6s-CAAX (cyan) and Ribeye b-mCherry (magenta) to label presynaptic ribbons in hair cells. An apical GCaMP6s-CAAX plane is used to examine mechanosensative Ca^{2+} influx in the mechanosensory hair bundles (a) while a plane at the base of the same neuromast can be used to monitor presynaptic Ca^{2+} influx at ribbons (b). (c) Similar to GCaMP6s-CAAX, red-shifted jRCaMP1a-CAAX (magenta) can measure apical or basal Ca^{2+} influx in hair cells. (d) Bongworri (cyan) can be used to detect membrane voltage changes in hair cells. (e) A double transgenic line expressing SypHy (cyan), an indicator of vesicle fusion and Ribeye b-mCherry (magenta) in hair cells can be used to monitor vesicle fusion at presynaptic ribbons. (f) Postsynaptic afferents expressing GCaMP6s-CAAX (cyan), along with expression of Ribeye b-mCherry (magenta) in hair cells enables monitoring of postsynaptic Ca^{2+} signals adjacent to presynaptic ribbons. (g) Cells expressing SypHy (cyan) and RGECO1 (magenta), a red cytosolic Ca^{2+} indicator enable dual monitoring of hair-cell Ca^{2+} and vesicle fusion. (h) A double transgenic with postsynaptic afferents expressing GCaMP6s-CAAX (cyan) and hair cells expressing RGECO1 (magenta) enable monitoring of pre- and post-synaptic Ca^{2+} activities respectively. Scale bar = 5 µm.

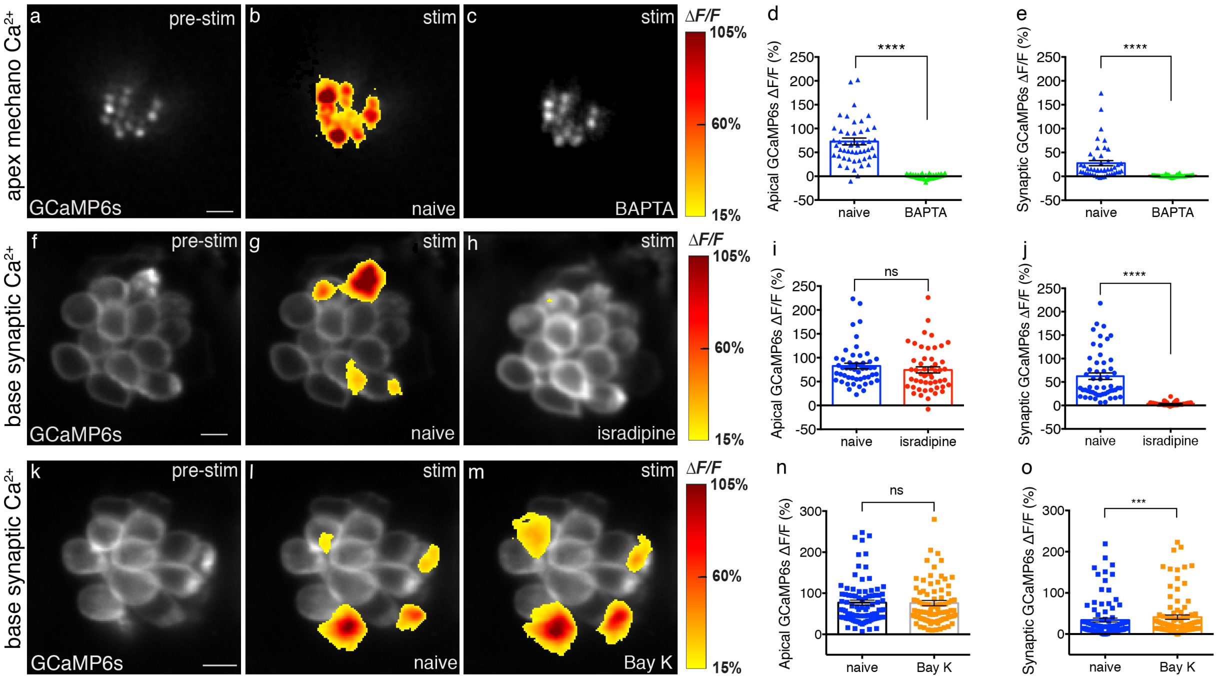

Supplementary Figure 2. **Effect of BAPTA, isradipine and Bay K on mechanosensitive Ca^{2} and presynaptic Ca^{2+}profiles of hair cells.** (a-c) Apical mechanosensitive bundle Ca^{2+}profiles of a single neuromast in response to a 2-s 5 Hz (anterior-posterior directed square wave) stimulus that activates all hair cells before (b) and after 5 mM BAPTA treatment (c). Spatial patterns of Ca^{2+} signals during stimulation are colorized according to the ΔF/F heat maps and superimposed onto the pre-stimulus baseline image (a). (d-e) Apical (naïve: 68.54 % ± 6.26; after BAPTA: 0.60 % ± 0.71, n = 46 cells, p < 0.0001) and presynaptic (naïve: 23.07 % ± 6.03; after BAPTA: 2.17 % ± 0.48, n = 46 cells, p < 0.0001) Ca^{2+} signals are both significantly decreased after 5 mM BAPTA treatment. (f-h) Presynaptic hair cell Ca^{2+}profiles of a single neuromast in response to a 2-s 5 Hz (anterior-posterior directed square wave) stimulus that activates all hair cells before (g) and after 10 µM isradipine treatment (h). Spatial patterns of Ca^{2+} signals during stimulation are colorized according to the ΔF/F heat maps and superimposed onto the pre-stimulus baseline image (f). (i-j) After 10 µM isradipine treatment, apical Ca^{2+} signals (naïve: 82.52 % ± 5.85; after isradipine: 74.31 % ± 6.37, n = 51 cells, p = 0.13) show no significant difference, but presynaptic Ca^{2+} signals (naïve: 62.40 % ± 7.05; after isradipine: 3.51 % ± .49, n = 51 cells, p < 0.0001) are significantly decreased. (k-m) Presynaptic hair cell Ca^{2+}profiles of a single neuromast in response to a 2-s 5 Hz (anterior-posterior directed square wave) stimulus that activates all hair cells before (l) and after 5 µM Bay K treatment (m). Spatial patterns of Ca^{2+} signals during stimulation are colorized according to the ΔF/F heat maps and superimposed onto the pre-stimulus baseline image (k). (n-o) After Bay K application, apical Ca^{2+} signals (naïve: 77.19 % ± 5.98; after Bay K: 76.01 ± 6.40, n = 94 cells, p = 0.51) show no significant difference, while presynaptic Ca^{2+} signals (naïve: 34.02 % ± 4.76; after bayK: 41.12 % ± 5.26, n = 94 cells, p = 0.0001) show significant increases. A Wilcoxon test was used in (d-e), (i-j), and (n-o), ***p < 0.001. ****p < 0.0001. Scale bars = 5 µm.

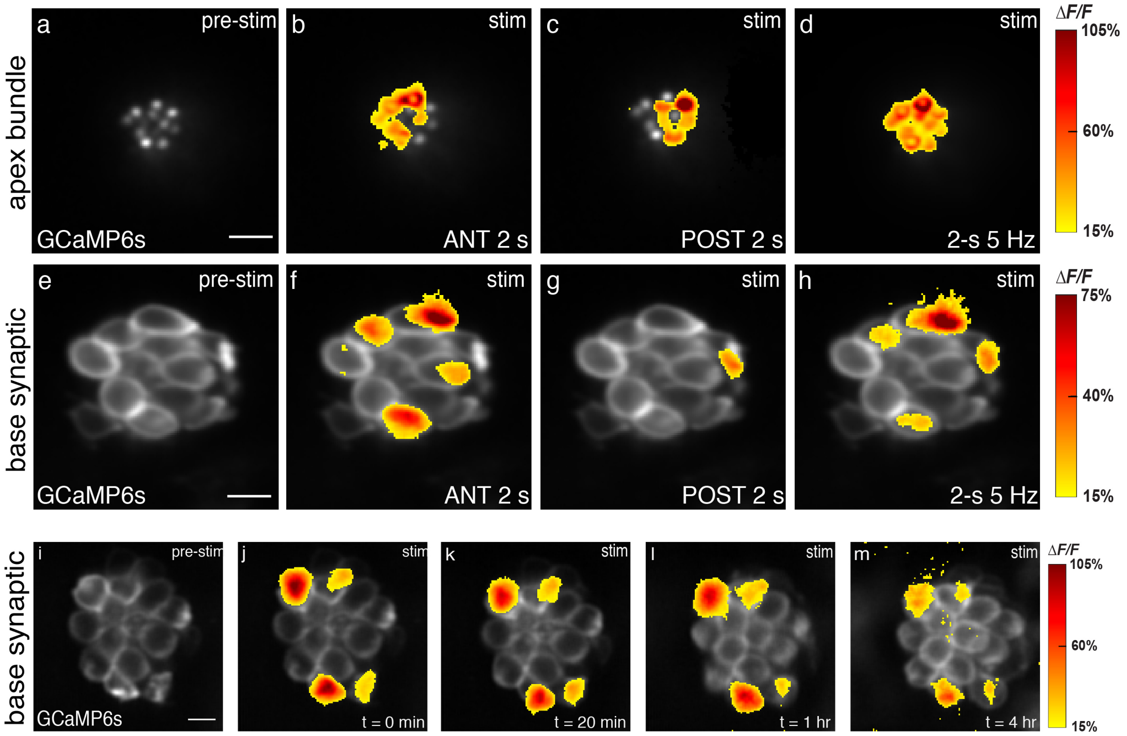

Supplementary Figure 3. **Two main stimulus types used in experiments; presynaptic responses are stable over time.** (a-d) Apical Ca^{2+} profiles in response to 2 s anterior stimulus (b), 2 s posterior stimulus (c) and 2-s 5 Hz frequency stimulus (d). (e-h) Preynaptic Ca^{2+} profiles from the same hair cells in (a-d), in response to 2 s anterior stimulus (f), 2 s posterior stimulus (g) and 2-s 5 Hz frequency stimulus (h). Spatial patterns of Ca^{2+} signals during stimulation are colorized according to the ΔF/F heat maps and superimposed onto pre-stimulus baseline images (a, e). (i-m) The same subset of cells had robust presynaptic Ca^{2+} influx over multiple trials with same stimulation after 20 min (k), 1 hr (l), and 4 hrs (m), n = 6 neuromasts. Spatial patterns of Ca^{2+} signals during stimulation are colorized according to the ΔF/F heat maps and superimposed onto pre-stimulus baseline image at each time point (ie: i for t = 0 min). Scale bars = 5 µm.

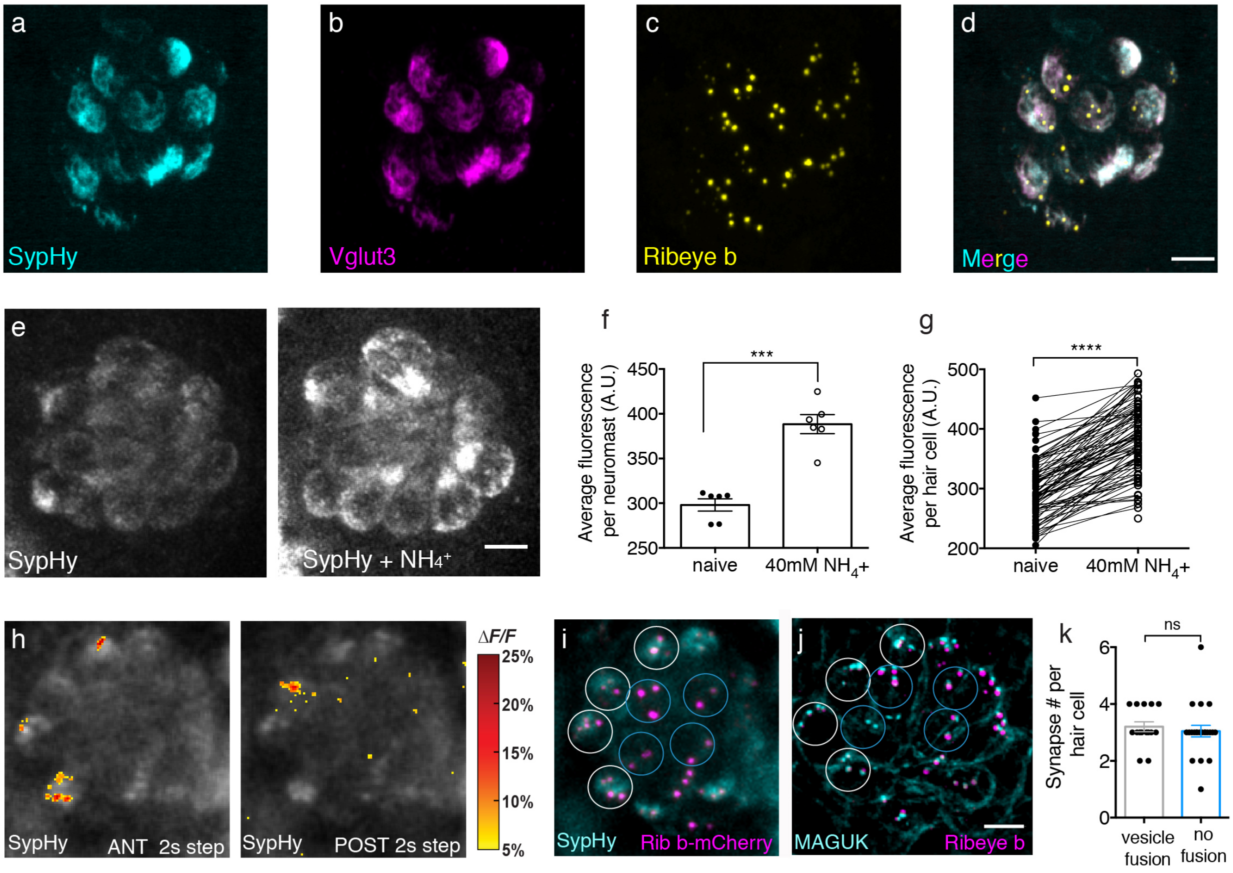

Supplementary Figure 4. **SypHy localizes to synaptic vesicles, and it is properly acidified; active and silent cells have the same number of synapses.** (a-d) Immunostaining of a representative neuromast expressing SypHy. Merged image (d) shows SypHy (a, cyan) colocalizes with Vglut3 (b, magenta) near Ribeye b labeled presynaptic ribbons (c, yellow). (e) Example neuromast demonstrating that baseline SypHy signals (e, left panel) increase when SypHy is deacidified after 40 mM NH_4Cl treatment (e, right panel). (f-g) Quantification of baseline SypHy intensities after 40 mM NH_4Cl treatment. The average SypHy signal per neuromast (f) is increased (naïve: 298.00 a.u. ± 6.87; after 40 mM NH_4^+: 388.40 a.u. ± 10.62, n = 6 neuromasts, p = 0.0007). All hair cells show an increase in baseline SypHy signal (g), (naïve: 297.40 a.u. ± 5.42; after 40 mM NH_4^+: 387.30 a.u. ± 6.53, n = 85 cells, p < 0.0001). (h) SypHy signals in response to 2-s anterior (h, left panel) and 2-s posterior (h, right panel) step stimuli that cumulatively activate all hair cells. Spatial patterns of SypHy signals during stimulation are colorized according to the ΔF/F heat maps and superimposed onto a pre-stimulus baseline image. (i) Live image of hair cells from the same neuromast as (h) expressing SypHy (cyan) and Ribeye b-mCherry (magenta). (j) The same neuromast as in (h-i), after immunostaining to label postsynaptic MAGUK (cyan) and presynaptic Ribeye b (magenta). White circles mark hair cells with vesicle fusion and blue circles indicate cells with no vesicle fusion. (k) Quantification of the number of synapses per individual hair cell based on MAGUK and Ribeye b staining show similar number of synapses between cells with (3.20 ± 1.18, n = 15 cells) and without (3.05 ± 0.20, n = 22 cells) vesicle fusion, p = 0.39, day

4-5. A paired t-test was used in (f-g), and a Mann-Whitney test was used in (k), ***p < 0.001, ****p < 0.0001. Scale bars = 5 µm.

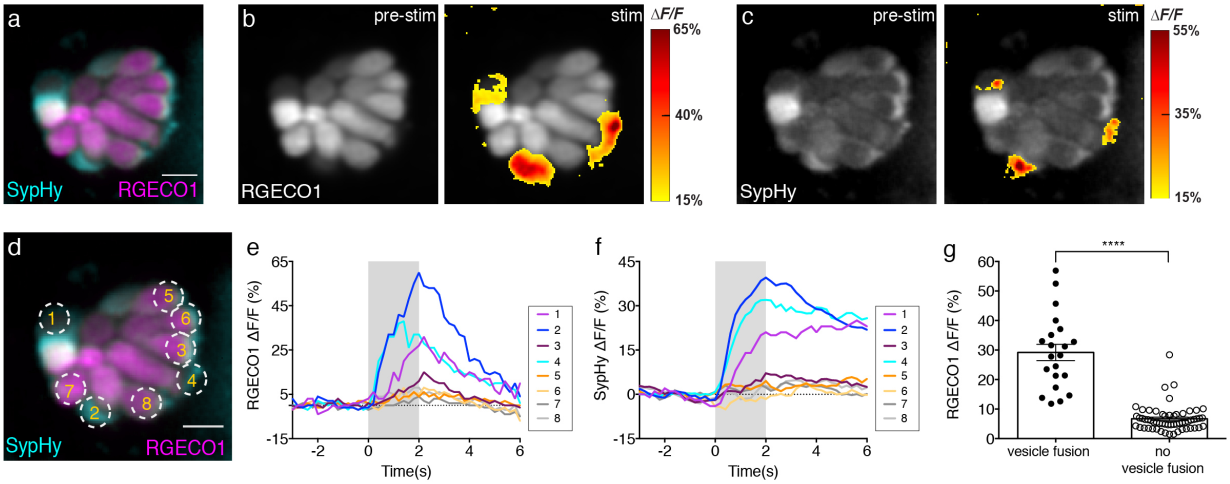

Supplementary Figure 5. **Hair cells with vesicle fusion correlate with strong cytosolic Ca^{2+} influx.** (a) Double transgenic neuromast co-expressing RGECO1 (magenta) and SypHy (cyan) in hair cells. (b-c) RGECO1 and SypHy responses acquired from the same neuromast organ during a 2-s 5 Hz (anterior-posterior directed square wave) stimulus that activates all hair cells. Spatial patterns of RGECO1 Ca^{2+} activities (b, right panel) or SypHy vesicle fusion (c, right panel) during stimulation are colorized according to the ΔF/F heat maps and superimposed onto pre-stimulus baseline grayscale image RGECO1 (b, left panel) or SypHy (c, left panel) image. (d) Combined color image of SypHy (cyan) and RGECO1 (magenta), with 8 hair cells outlined with ROIs (3 µm). (e-f) Temporal curves of RGECO1 Ca^{2+} signals (e) and SypHy vesicle fusion signals (f) from the 8 ROIs drawn in (d). Hair cells (cells 1, 2, 4) with stronger Ca^{2+} influx (e) also have detectable vesicle fusion (f). (g) RGECO1 Ca^{2+} signal magnitude is greater in hair cells with (ΔF/F, 29.18 % ± 2.75, n = 21 cells) than without (ΔF/F, 6.70 % ± 0.58, n = 58 cells) detectable SypHy signals, p< 0.0001. A Mann Whitney test was used in (g), **** p < 0.0001. Scale bars = 5 µm.

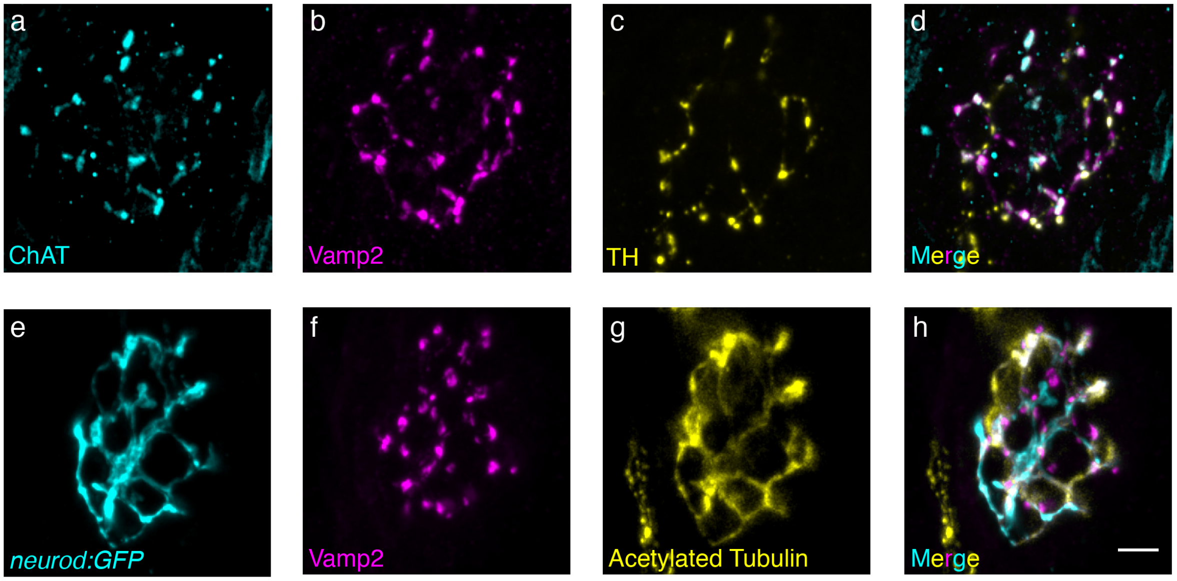

Supplementary Figure 6. **Dopaminergic and cholinergic efferent neurons innervate lateral-line neuromasts.**

(a-d) Neuromast immunostaining of ChAT (cyan, a) and TH (yellow, c) label cholinergic and dopaminergic efferents respectively. Colocalization of Vamp2 (magenta, b), a neuronal presynaptic marker, with ChAT and TH (d) demonstrates that both types of efferents make presynaptic contacts on or near neuromasts. (e-h) Neuromast afferents expressing *neurod:GFP* (cyan, e) demonstrate that cumulatively, Acetylated tubulin (yellow, g) and Vamp2 (magenta, f) immunostaining can be used to label afferent and efferent innervation respectively. Scale bar = 5 µm.

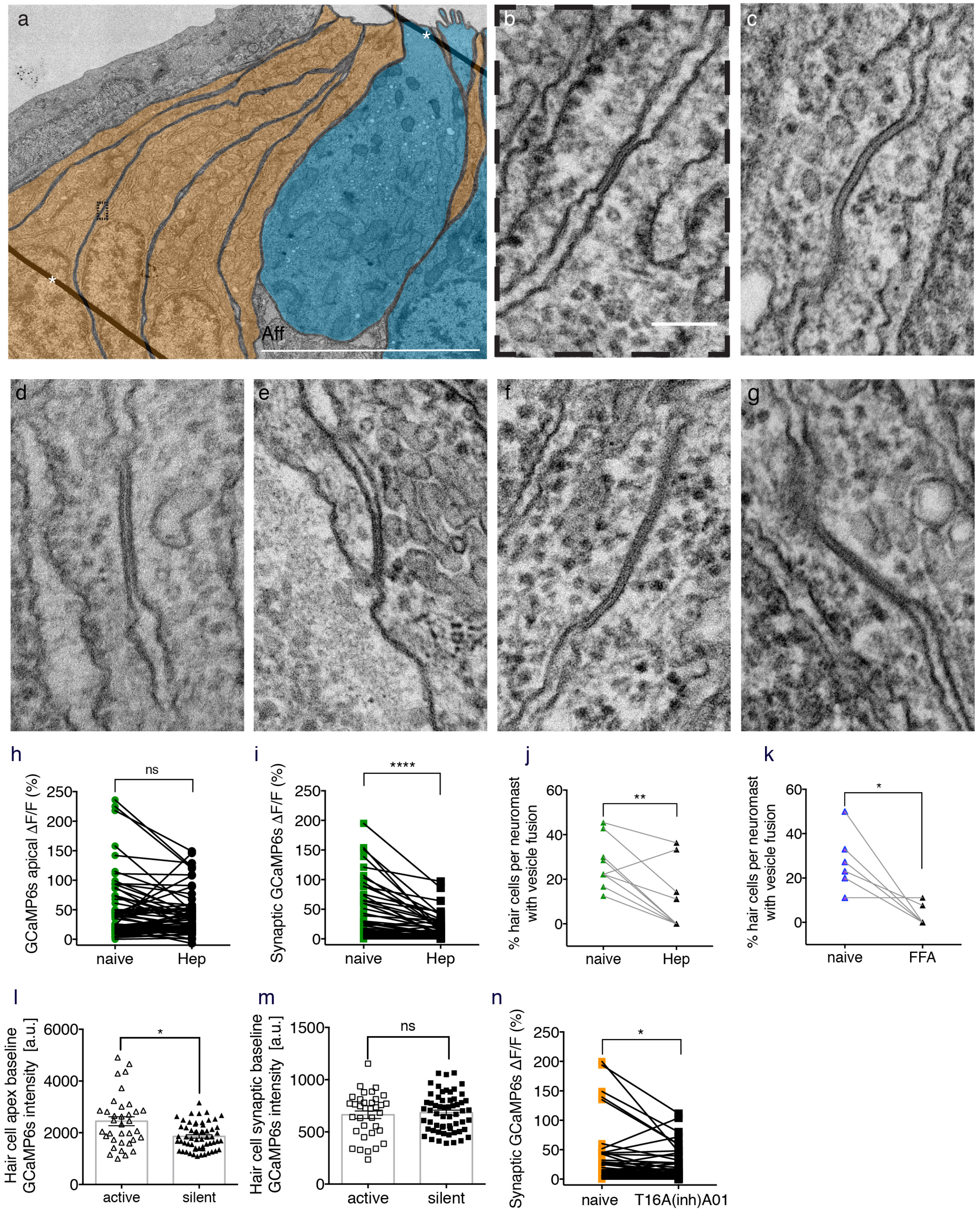

Supplementary Figure 7. **Gap junctions between supporting cells are important for presynaptic function in hair cells.** (a) A representative transmission electron microscopy (TEM) image of a neuromast with hair cells colorized in blue and supporting cells in orange. (b)

Enlarged image of the dashed box in (a) reveals a cross section through a gap junction between two supporting cells. (c-g) Additional examples of a gap junction between two supporting cells. (h) Mechanosensitive Ca^{2+} signals show no significant differences before and after 3 mM heptanol treatment (naïve: 48.69 % ± 6.26; heptanol: 39.82 % ± 4.37, n = 68 cells, p = 0.07). (i) In the same neuromast organs as (h), presynaptic Ca^{2+} signals are significantly decreased after 3 mM heptanol treatment (naïve: 33.13 % ± 5.27: heptanol: 13.49 % ± 2.26, n = 70 cells, p < 0.0001). (j, k) The percentage of hair cells with vesicle fusion per neuromast is significantly decreased after 3 mM heptanol treatment (naïve: 26.46 % ± 3.35; heptanol: 10.62 % ± 4.42, n = 10 neuromasts, p = 0.003) and 25 µM FFA treatment (naïve: 27.41 % ± 5.42; FFA: 3.31 % ± 2.02, n = 6 neuromasts, p = 0.02). (l) Using GCaMP6s, there are slight differences in the resting bundle Ca^{2+} levels in active (2446 a.u. ± 171.9, n = 35 cells) versus silent hair cells (1860 a.u. ± 66.74, n = 59 cells), p = 0.02. (m) In the same neuromast organs as (l), there are no significant differences in the resting presynaptic Ca^{2+} levels between active (664.3 a.u. ± 35.84, n = 35 cells) and silent hair cells (685.4 a.u. ± 23.58, n = 59 cells), p = 0.81. (n) Using 10 µm of the chloride channel blocker T16(inh)-A01, there was a significant decrease in presynaptic Ca^{2+} signals (naïve: 24.67 % ± 5.32: T16(inh)-A01: 16.62 % ± 3.00, n = 66 cells, p = 0.02). Asterisks in (a) indicate lines that are folds in the section. Aff = afferent process. A Wilcoxon test was used in (h-i, n), a paired t-test in (j-k), and a Mann-Whitney test in (l-m), *p <0.05, **p <0.01, ****p <0.0001, Scale bar = 5 µm in (a) and it is 100 nm in (b-f) .
